# Molecular Dynamics-Guided Repositioning of FDA-Approved Drugs for PD-L1 Inhibition with In Vitro Anticancer Potential

**DOI:** 10.3390/ijms26104497

**Published:** 2025-05-08

**Authors:** Dejun Jiang, Myoung-Schook Yoou, Sungjoon Cho, Youngjin Choi

**Affiliations:** 1 Department of Environmental Engineering, Hoseo University, Asan 31499, Republic of Korea; jdejun@hotmail.com; 2 Eulji Medi-Bio Research Institute, Eulji University, Daejeon 34824, Republic of Korea; 20250025@eulji.ac.kr; 3 Department of Bio-Applied Toxicology, Hoseo University, Asan 31499, Republic of Korea; pertinencyto@gmail.com; 4 Department of Food Science & Technology, Hoseo University, Asan 31499, Republic of Korea

**Keywords:** PD-L1, drug repositioning, molecular dynamics simulation, cancer, virtual screening

## Abstract

Programmed death-ligand 1 (PD-L1) is a crucial immune checkpoint protein that tumors often exploit to evade immune surveillance. This study systematically screened a library of 1031 FDA-approved drugs using a high-throughput molecular dynamics approach to identify potential inhibitors targeting PD-L1. From this screening, five promising compounds—vorapaxar, delafloxacin, tenofovir disoproxil, pivmecillinam, and fursultiamine—showed significant binding affinities to PD-L1 and demonstrated cytotoxic activity against A549 lung tumor cells. These candidates were further evaluated through extended molecular dynamics simulations lasting up to 150 ns to assess their structural stability, residue fluctuations, and binding free energy. Among the identified compounds, pivmecillinam demonstrated the most favorable results, exhibiting stable binding interactions and a binding free energy of −18.01 kcal/mol, comparable to that of the known PD-L1 inhibitor BMS-1. These findings suggest that pivmecillinam has promising immunomodulatory potential and could serve as a candidate for further development in cancer immunotherapy. Overall, this study underscores the value of integrating high-throughput MD and experimental approaches for drug repositioning to identify novel therapeutic agents.

## 1. Introduction

Programmed death-ligand 1 (PD-L1) is a membrane protein expressed in various tumor cell types that binds to its receptor, programmed death-1 (PD-1), primarily found on activated T cells [1,2,3]. The PD-1/PD-L1 interaction plays a crucial role in immune evasion by tumors, suppressing T-cell responses and allowing cancer progression [4,5]. Preclinical studies have demonstrated that blocking this interaction enhances antitumor immunity, leading to the development of immune checkpoint inhibitors (ICIs) targeting PD-1 and PD-L1 [6]. Various monoclonal antibodies have revolutionized cancer treatment, showing significant efficacy in clinical trials for various solid and hematological malignancies [7]. Notably, the FDA-approved PD-1 inhibitors nivolumab and pembrolizumab, as well as PD-L1 inhibitors such as atezolizumab, have substantially improved patient outcomes [8,9,10]. Furthermore, immune checkpoint inhibitors targeting the PD-1/PD-L1 pathway are authorized for treating several cancers, including classical Hodgkin lymphoma and head and neck squamous cell carcinoma [11]. However, despite their success, antibody-based ICIs present several challenges, including high production costs, immune-related adverse effects, and limited tumor penetration [12]. These limitations have driven interest in small-molecule inhibitors as a promising alternative [13]. Yet, traditional drug discovery is a lengthy, high-risk process requiring substantial investment, leading to a growing preference for drug repositioning, which repurposes existing drugs for new therapeutic applications [14].

Drug repositioning offers a cost-effective strategy by leveraging available data on pharmacokinetics, safety, and manufacturing [15]. Compared to de novo drug development, repositioning significantly reduces both time and costs while mitigating the risks associated with drug approval [16]. The three primary approaches to drug repositioning—computational, bio-experimental, and mixed methods—have gained widespread adoption [17]. Computational approaches, in particular, enable the systematic identification of novel drug-target interactions by integrating large-scale biological and chemical data, fostering innovation in pharmaceutical development [18].

Computational approaches for drug repositioning can reveal intricate connections among drugs, targets, disease genes, and illnesses within a systems framework [19]. Mahgoub and his team pinpointed two commercially available drug-like compounds that target TMPRSS2 using pharmacophore modeling, homology modeling, and molecular docking methods [20]. Cava et al. utilized public gene expression profile datasets to discover new functions and mechanisms associated with ACE2-related genes, which are believed to facilitate SARS-CoV-2 entry into cells [21]. Given the lengthy and costly process of developing a candidate drug into an approved treatment, adopting computational approaches—including virtual screening, docking, molecular dynamics (MD) simulation, and binding free energy evaluation—provides a practical alternative for identifying potential drug candidates from compound libraries [22].

**Figure 1:**
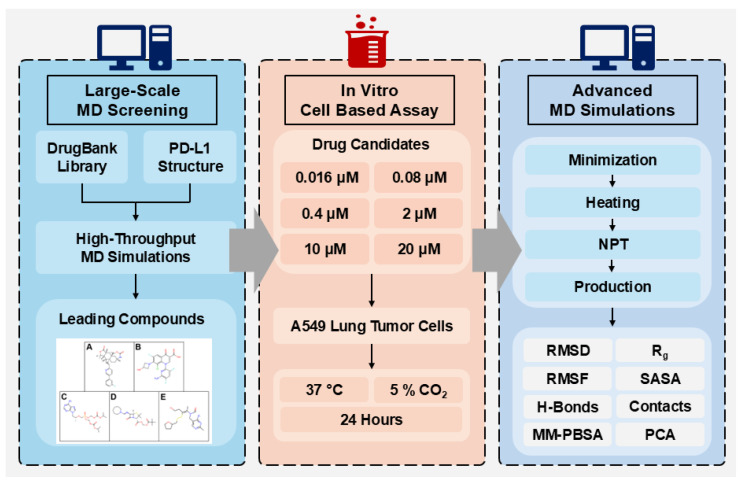
Flowchart of the current study illustrating the three experimental phases: large-scale molecular dynamics screening, in vitro MTT assay, and advanced long-term molecular dynamics simulations.

In this study, a high-throughput molecular dynamics framework was applied to identify FDA-approved drugs capable of inhibiting PD-L1 [23]. A total of 1031 DrugBank compounds were screened based on their binding affinity to the PD-L1 receptor, followed by MD simulations to assess stability and interaction strength. Among these, the top five compounds demonstrated strong PD-L1 inhibition and were further validated through in vitro MTT assays, revealing significant anticancer effects against A549 lung cancer cells. This study highlights a computational-experimental hybrid approach, demonstrating the potential of drug repositioning to uncover novel anticancer agents targeting immune checkpoint pathways, as shown in Figure 1.

## 2. Results and Discussions

### 2.1. Large-Scale Molecular Dynamics Screening

Several rounds of in situ virtual screening have been performed for discovering PD-L1 inhibitors by other studies. Sun et al. and Kamal et al. conducted pharmacophore-based screenings using the DrugBank small molecule library and SPECS library and recommended using daclatasvir, compound H2 and H4 as PD-L1 inhibitors [24,25]. In this study, high-throughput molecular dynamics simulations were conducted on 1031 FDA-approved small-molecule drugs from the DrugBank library. This method offers advantages over conventional docking mechanisms in predicting the affinities between proteins and ligands due to its dynamic calculation approach, rather than relying on single statistical results, and also skips the long-term development period of developing new compounds [26]. Both generalized born (GB) and Poisson–Boltzmann (PB) models from the MM-PBSA module of AMBERTools 24 were performed to predict the binding free energy of these candidates [27].

In the GB-model results, 35 chemicals exhibited a binding free energy of −40 kcal/mol, while in the PB-model, 22 chemicals met this threshold, resulting in a total of 43 chemicals with the potential to achieve −40 kcal/mol. Among these, only two have received FDA approval for targeting tumors: brigatinib and palbociclib. The other 39 chemicals are newly identified potential anticancer agents that inhibit PD-L1.

**Figure 2:**
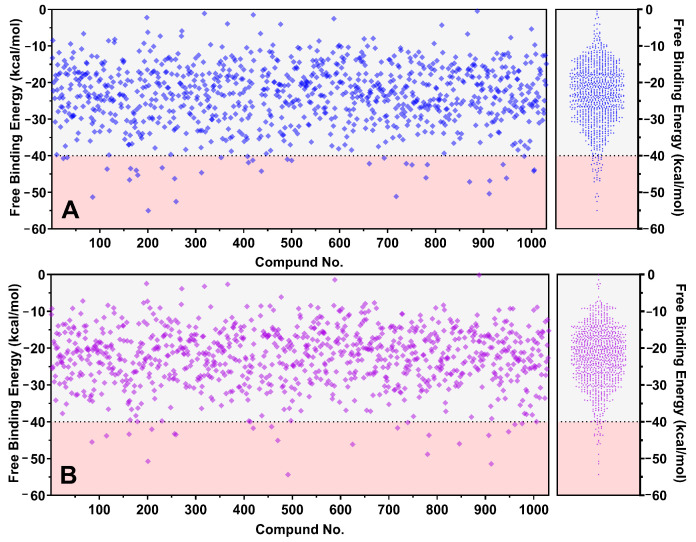
Binding free energy distributions from MM-GB(PB)SA calculations using (**A**) the GB model and (**B**) the PB model for 1031 FDA-approved drugs during extensive MD pre-screening, with red zones representing the screening threshold of −40 kcal/mol.

As presented in Figure 2, MM-(GB)PBSA results using the GB model had a maximum value of −55.02 kcal/mol, while the minimum was −0.47 kcal/mol, and the average was −23.39 ± 8.33 kcal/mol. The PB model, which utilizes an advanced algorithm, showed the highest affinity of −54.36 kcal/mol, with a minimum of −0.13 kcal/mol and an average of −22.41 ± 7.81 kcal/mol.

The generalized born model is recognized for its high-speed calculations while sacrificing accuracy with its approximation function [28]. In contrast, the Poisson–Boltzmann model, which calculates more rigorously, is better suited for complex systems, although it is significantly more computationally expensive than the GB model [29]. Notably, the GB model closely aligns with the PB model despite its significantly faster computation and simpler mechanism, demonstrating a high performance-to-cost ratio. This is attributed to a straightforward system consisting of PD-L1 and a basic ligand, missing the advantages shown by the PB model, which deals with more complex scenarios [30].

**Figure 3:**
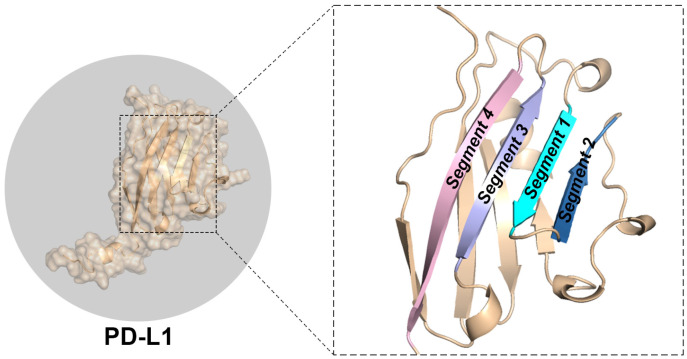
The three-dimensional structure of PD-L1, highlighting its major binding β-sheets, specifically Segments 1 to 4.

The binding regions of PD-L1 are well-defined and comprise four parallel β-sheets: Segment 1 (Residue No. 38–43), Segment 2 (Residue No. 46–52), Segment 3 (Residue No. 94–101), and Segment 4 (Residue No. 105–115), as shown in Figure 3. These binding sites were previously examined by Guo et al. in 2021 and are further validated by analyzing the simulation trajectory through large-scale MD simulations in this study [31].

During the initial in silico screening, 41 compounds approved by the FDA were found to be potential PD-L1 inhibitors, each exhibiting a predicted binding free energy lower than −40 kcal/mol, as detailed in Table S1. 14 of these agents were available commercially and moved on to a rapid primary in vitro viability screen prior to the formal MTT assays. In this assay, illustrated in Figure S1, A549 lung carcinoma cells were treated with each compound at a concentration of 2 µM for 24 h. Compounds that decreased cell viability to below 75% of the untreated control were selected, resulting in 7 preliminary hits. Five of these agents had no prior oncological use, while two were already established anticancer medications.

**Figure 4:**
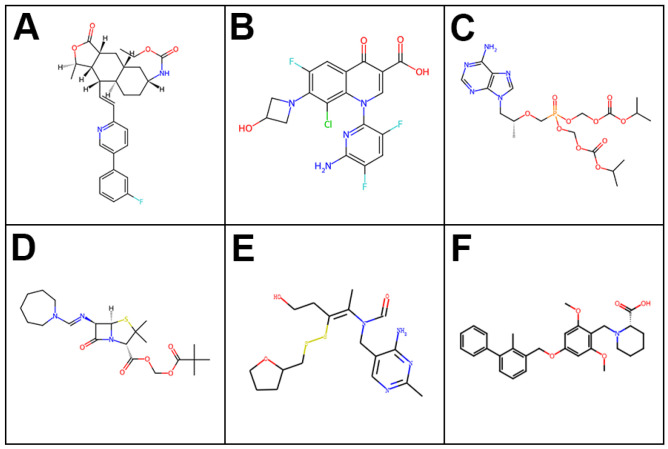
Chemical structures of the five newly identified anticancer candidates along with the reference PD-L1 inhibitor: (**A**) vorapaxar, (**B**) delafloxacin, (**C**) tenofovir disoproxil, (**D**) pivmecillinam, (**E**) fursultiamine, and (**F**) BMS-1.

Based on their results in the primary viability screen and their recognized clinical uses, five compounds—vorapaxar, delafloxacin, tenofovir disoproxil, pivmecillinam, and fursultiamine—were chosen for MTT evaluation and underwent extended molecular dynamics simulations. The chemical structures of these candidates, along with the reference PD-L1 inhibitor BMS-1, are shown in Figure 4.

### 2.2. MTT Cell Assays for Leading FDA-Approved Drugs

The MTT assay, a colorimetric method, is used to assess cellular metabolic activity and is frequently applied to investigate the cytotoxicity and effectiveness of cancer treatments [32]. The A549 lung adenocarcinoma cell line is a preferred choice for evaluating the cytotoxic impacts of drug candidates in early screening phases [33]. In this study, the MTT assay was performed on A549 cells to identify a potential anti-lung cancer feature of five compounds. All candidates demonstrated effectiveness in the assay conducted at dynamic concentrations ranging from 0.016 μM to 20 μM.

**Figure 5:**
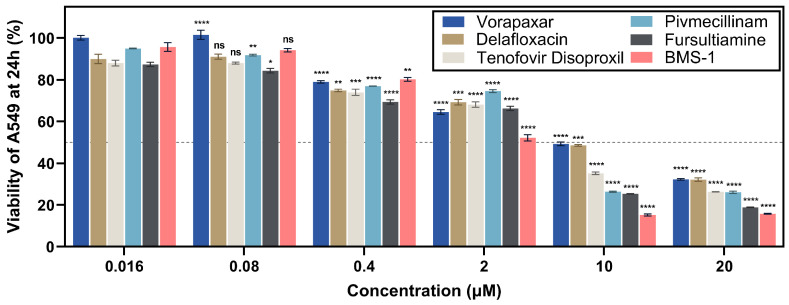
Viability of A549 treated with essential compounds, measured using the MTT assay after 24 h; **** *p* < 0.0001, *** *p* < 0.001, ** *p* < 0.01, * *p* < 0.05, and ns *p* > 0.05, based on Welch’s *t*-test, with the dashed line representing 50% cell viability.

As illustrated in Figure 5, none of the tested compounds produced notable cytotoxicity at the lowest concentration of 0.016 µM. At 0.08 µM, pivmecillinam and fursultiamine induced statistically significant (*p* < 0.05) minor reductions in A549 cell viability. Starting at 0.4 µM, every candidate compound meaningfully decreased viability, with the majority lowering cell survival to under 80%. At 2 μM, BMS-1, a known PD-L1 inhibitor, was the first to approach its half-maximal inhibitory concentration. When tested at 10 μM, all candidate compounds were capable of killing over 50% of tumor cells, while BMS-1 exhibited the highest cytotoxic potential with a cell viability of 15.2%. Fursultiamine and pivmecillinam followed, with cell viabilities of 25.4% and 26.4%, respectively. Vorapaxar and delafloxacin exhibited milder cytotoxic effects, with viabilities of 49.3% and 48.6%, respectively. Tenofovir disoproxil produced an intermediate result, demonstrating 35.2% viability at this concentration. At the highest tested dose of 20 μM, all candidate agents displayed significant cytotoxic activity against A549 cells, with fursultiamine reaching 18.9% cell viability, closely matching the effect of BMS-1 developed by Bristol Myers Squibb.

Prior studies have demonstrated that specific candidate compounds, including vorapaxar, fursultiamine, and tenofovir disoproxil, can influence A549 cell viability through distinct mechanisms. Vorapaxar has been reported to suppress TGFβ-enhanced PAR-1 signaling, which is implicated in tumor progression, while fursultiamine is associated with the inhibition of reactive oxygen species, a key factor in cancer cell survival [34,35]. Additionally, high concentrations of tenofovir disoproxil have been shown to reduce A549 cell viability, suggesting potential cytotoxic effects [36]. Although these findings indicate that these compounds may exhibit antiproliferative properties, no studies have specifically investigated their role as PD-L1 inhibitors or their potential to protect immune cells from PD-1-mediated suppression. Given that PD-L1 inhibition can enhance immune responses against tumors, further exploration of these compounds in this context is warranted.

While the MTT assay effectively demonstrated the cytotoxic effects of the candidate compounds on tumor cells, relying solely on this cell-based assay has limitations. This approach faced difficulties in quantifying the binding energy between candidates and PD-L1. Future cellular evaluations might be enhanced by incorporating methods such as surface plasmon resonance (SPR) or isothermal titration calorimetry (ITC) [37]. These techniques could validate the results of this study and offer additional insights into drug–PD-L1 interactions.

By performing long-term molecular dynamics simulations and accompanying post-MD analyses, drug repositioning strategies play a crucial role in evaluating the additional action mechanisms of the candidates in this study. These methods will assess whether the chosen compounds can bind to PD-L1 similarly to BMS-1, one of the few reference PD-L1 inhibitors, beyond their recognized FDA-approved uses [38]. The insights gained from this comparative binding analysis could indicate prolonged, inhibitory interactions between the candidate compounds and PD-L1, presenting a promising therapeutic pathway for immunomodulatory intervention. Additionally, such results would enhance the understanding of the structural elements essential for PD-L1 inhibition, guiding future drug design aimed at improving cancer immunotherapies.

### 2.3. Post-MD Analysis of Leading Candidates

#### 2.3.1. RMSD Analysis

**Figure 6:**
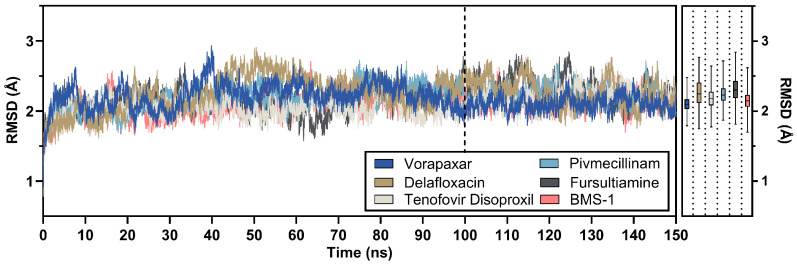
RMSD of 150 ns advanced molecular dynamics simulations for key FDA compounds and BMS-1, with nested average RMSD values shown via box and whisker plots derived from the final 50 ns of simulation, and the dashed line indicating the start of the analysis period.

The root-mean-square deviation (RMSD) of candidates was analyzed to assess the overall stability of the PD-L1 and candidate complexes. In the final 50 ns phase deemed reliable for analysis, all candidate compounds exhibited stable behavior compared to the initial 100 ns period. As shown in Figure 6, vorapaxar exhibited the lowest average RMSD value at 2.10 ± 0.10 Å. This was followed by BMS-1, the PD-L1 inhibitor, with an RMSD of 2.15 ± 0.12 Å. Tenofovir disoproxil, pivmecillinam, and delafloxacin had moderate RMSD values of 2.18 ± 0.14 Å, 2.24 ± 0.13 Å, and 2.26 ± 0.19 Å, respectively. Fursultiamine had a comparatively higher RMSD of 2.313 ± 0.180 Å, indicating greater structural fluctuations.

Generally, the RMSD ranged from 1.8 Å to 2.6 Å, with a general average of 2.21 Å. This indicates that the leading candidate drugs representing their target groups formed a strong bond with PD-L1 and maintained this relationship over a long period, similar to the reference chemical.

#### 2.3.2. RMSF Analysis

**Figure 7:**
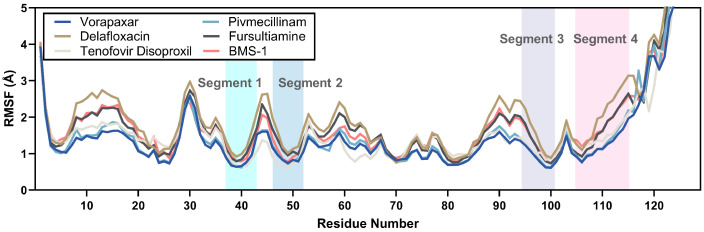
RMSF values derived from advanced molecular dynamics simulations of selected key FDA-approved compounds and the reference chemical.

Throughout the 150 ns simulation period, the root-mean-square fluctuation (RMSF) values of the five candidate compounds were analyzed to gather insights into residue-level stability. The RMSF values generally ranged from 0.5 Å to 4.0 Å, with an average of 1.49 Å, while residues beyond position 120 were excluded due to their minimal reference value. As exhibited in Figure 7, vorapaxar demonstrated the lowest average RMSF of 1.27 ± 0.57 Å, which corresponded with its RMSD performance. Pivmecillinam, tenofovir disoproxil, and fursultiamine showed moderate average RMSF values of 1.32 ± 0.59 Å, 1.39 ± 0.50 Å, and 1.59 ± 0.62 Å, respectively. In contrast, delafloxacin and BMS-1 had relatively higher averages of 1.80 ± 0.71 Å and 1.59 ± 0.60 Å, respectively.

Among the four beta-sheet segments of interest (Segments 1–4), all candidate compounds displayed low RMSF values. The average RMSF values across these segments were 1.00 ± 0.37 Å, 1.06 ± 0.31 Å, 1.17 ± 0.44 Å, and 1.60 ± 0.56 Å, respectively. Segment 1 (residues 35–43) indicated the smallest fluctuations, while Segment 4 (residues 105–115), positioned closer to the protein’s C-terminus, presented comparatively higher RMSF values, which align with findings in later parts of this study.

#### 2.3.3. R_g_ and SASA Analysis

**Figure 8:**
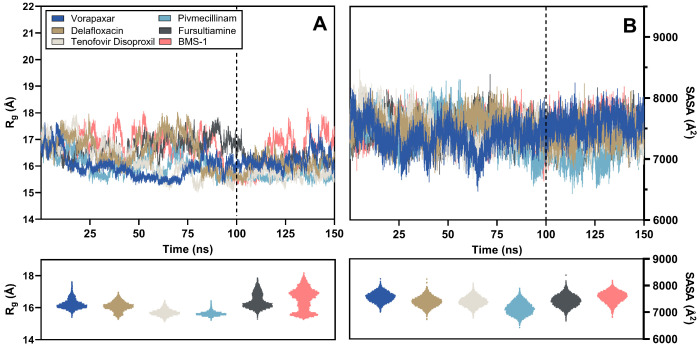
(**A**) R_g_ and its distribution obtained from long-term molecular dynamics simulations of essential FDA-approved compounds and BMS-1; (**B**) SASA and its arrangement from simulations of key FDA-approved compounds and the reference chemical.

In the final 50 ns, chosen for its comparative stability against the initial 100 ns, the radius of gyration (R_g_) for all candidate compounds was analyzed to assess the overall compactness of the PD-L1 complex. As illustrated in Figure 8, pivmecillinam and tenofovir disoproxil showed similar average R_g_ values of 15.63 ± 0.14 Å and 15.70 ± 0.20 Å, respectively, with minor fluctuations. Delafloxacin, fursultiamine, and vorapaxar displayed intermediate R_g_ values of 16.07 ± 0.29 Å, 16.17 ± 0.25 Å, and 16.22 ± 0.31 Å, respectively. BMS-1, notably, had a higher average R_g_ of 16.53 ± 0.67 Å, indicating more variability.

The same analytical method was used to evaluate solvent-accessible surface area (SASA), which examined the exposure level of the structure to the solvent. The SASA results indicated a consistently stable profile in the final 50 ns, demonstrating greater reliability than in the preceding 100 ns. Pivmecillinam had the lowest average SASA at 7141.52 ± 226.86 Å^2^, consistent with its R_g_ findings. Fursultiamine, delafloxacin, and tenofovir disoproxil presented moderate average SASA values of 7392.33 ± 208.63 Å^2^, 7397.14 ± 187.05 Å^2^, and 7405.88 ± 183.54 Å^2^, respectively. Conversely, BMS-1 and vorapaxar exhibited larger SASA values of 7571.89 ± 206.47 Å^2^ and 7573.27 ± 178.52 Å^2^, respectively, which correlate with their higher average R_g_ values.

#### 2.3.4. Hydrogen Bonds Analysis

**Figure 9:**
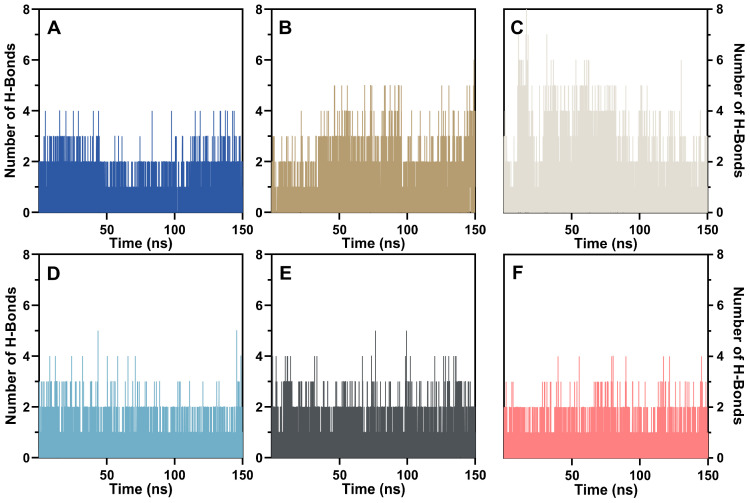
Number of hydrogen bonds obtained from advanced molecular dynamics simulations of important FDA compounds and the inhibitor: (**A**) vorapaxar, (**B**) delafloxacin, (**C**) tenofovir disoproxil, (**D**) pivmecillinam, (**E**) fursultiamine, and (**F**) BMS-1.

Hydrogen bonding is another major contributor to the binding affinity between a protein and its ligands. Overall, hydrogen bond numbers during 150 ns simulations indicated that the candidate compounds, as well as BMS-1, formed fewer hydrogen bonds per frame with PD-L1. As shown in Figure 9, tenofovir disoproxil exhibited the highest average number of hydrogen bonds per frame, 0.87, and reached a maximum of 8 H-bonds with PD-L1. Delafloxacin, vorapaxar, and fursultiamine displayed intermediate H-bonds of 0.58, 0.39, and 0.36 per frame, respectively. Pivmecillinam and BMS-1 yielded 0.23 and 0.21 per frame, respectively, suggesting that BMS-1 does not depend heavily on hydrogen bonding to achieve stable binding with PD-L1.

As shown in Table 1, distinct preferences for donor or acceptor roles were observed among these compounds. Vorapaxar, delafloxacin, and fursultiamine principally acted as donors by contributing hydrogen atoms to TYR40 and MET99. In contrast, tenofovir disoproxil, pivmecillinam, and BMS-1 primarily served as acceptors, interacting with ARG109 and GLN50. Among PD-L1 residues, ARG109 in Segment 4 emerged as the most influential donor, providing 25.15% of the hydrogen atoms to tenofovir disoproxil and 6.67% to vorapaxar. This outcome corresponded to tenofovir disoproxil’s ability to establish the highest number of hydrogen bonds with PD-L1. MET99 in Segment 3 constituted the most active acceptor residue, enabling the formation of 7.54%, 5.08%, and 2.73% of total hydrogen bonds with delafloxacin, fursultiamine, and pivmecillinam, respectively. BMS-1 formed hydrogen bonds only with GLN50 in Segment 2, both as donor and acceptor, albeit at relatively low levels. These findings further support the conclusion that BMS-1 does not rely extensively on hydrogen bonding for close binding with PD-L1.

#### 2.3.5. Contact Numbers Analysis

Throughout the 150 ns simulation, the contact frequencies among PD-L1 residues located within 4.5 Å of each candidate compound were assessed to evaluate the exposure level of each critical residue to the compounds. Fursultiamine had the highest average contact frequency at 49.90 per frame. Pivmecillinam, BMS-1, and tenofovir disoproxil had intermediate frequencies of 31.28, 29.28, and 26.45 per frame, respectively, while vorapaxar and delafloxacin had lower frequencies of 15.98 and 17.51 per frame.

**Figure 10:**
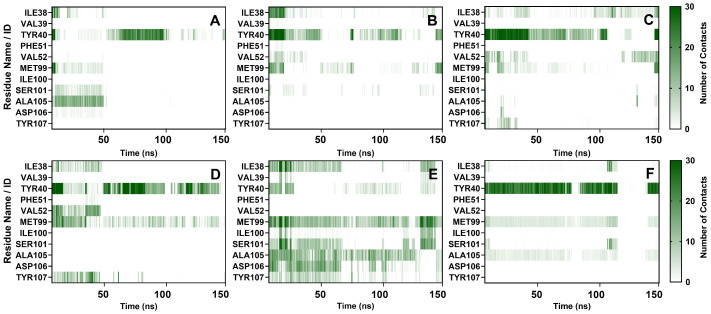
Number of contacts between leading candidates and PD-L1: (**A**) vorapaxar, (**B**) delafloxacin, (**C**) tenofovir disoproxil, (**D**) pivmecillinam, (**E**) fursultiamine, and (**F**) BMS-1.

As shown in Figure 10, specific PD-L1 residues made significant contributions. TYR40 in Segment 1 accounted for over 50% of total contacts for all candidates, except Fursultiamine, and contributed 75.57% of the contacts for BMS-1, serving as the primary binding site. The interactions were mainly composed of alkyl interactions and hydrogen bonding, with TYR40 acting as a hydrogen acceptor. MET99 in Segment 3 was the second-largest contributor to delafloxacin, pivmecillinam, and BMS-1, and it became the main binding site for fursultiamine. MET99 was primarily engaged in alkyl and π-alkyl interactions while also serving as a significant hydrogen acceptor. Other critical residues were involved in contacts to varying degrees, with some losing contact over time and others forming new interactions as the simulation progressed.

#### 2.3.6. Binding Free Energy Analysis

The MM-PBSA method was used to evaluate binding free energies in this study. These candidate compounds were chosen from a library of 1031 FDA-approved drugs based on preliminary screenings, indicating a potentially improved affinity for Programmed Death-Ligand 1. As shown in Table 2, the PD-L1 inhibitor BMS-1 exhibited the most favorable binding free energy of −27.70 ± 4.44 kcal/mol. Among the candidates, fursultiamine came closest with a value of −19.23 ± 3.94 kcal/mol. Other compounds showed intermediate binding free energies: pivmecillinam at −18.01 ± 3.94 kcal/mol, vorapaxar at −17.97 ± 3.88 kcal/mol, and tenofovir disoproxil at −17.55 ± 7.50 kcal/mol, while delafloxacin recorded −12.37 ± 4.00 kcal/mol.

Van der Waals (VDW) interactions were the main contributors to vacuum-phase binding across all candidates, complemented by electrostatic free energy (EEL). Delafloxacin exhibited a notable positive electrostatic offset, which diminished its overall binding free energy. While advantageous vacuum contributions were observed in all instances, solvation energies varied in their level of impact. BMS-1 displayed strong overall binding due to its significant vacuum-phase interactions and relatively consistent performance in solvation. Fursultiamine demonstrated a similar mechanism, contributing to its favorable net binding free energy. Tenofovir disoproxil also had a robust vacuum contribution but faced a considerable energy offset in solvation, leading to a reduced net binding free energy.

**Figure 11:**
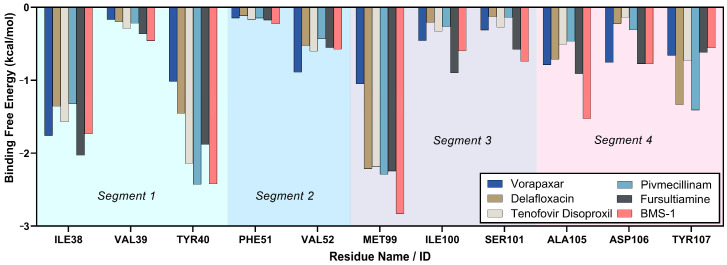
Per-residue binding free energy of Segments 1–4, derived from advanced molecular dynamics simulations of key FDA-approved compounds and the reference chemical.

Among the key PD-L1 residues, MET99 in Segment 3 accounted for 21.07% of the binding free energies, as shown in Figure 11. It was followed by TYR40 and ILE38 in Segment 1, contributing 18.65% and 16.07%, respectively. Other residues in different segments each contributed less than 10%. A comparison by segment revealed that Segment 1 had the highest contribution at 37.52%, followed by Segment 3, Segment 4, and Segment 2 in that order. BMS-1 captured substantial binding energy from nearly all critical residues, while fursultiamine and pivmecillinam showed notable contributions as well. Fursultiamine obtained most of its binding energy from ILE38 and ILE100, surpassing BMS-1’s contribution at those residues. In contrast, pivmecillinam primarily drew its major contributions from TYR40 and TYR107, exceeding the contributions from the other compounds at those locations.

#### 2.3.7. Principal Component Analysis

**Figure 12:**
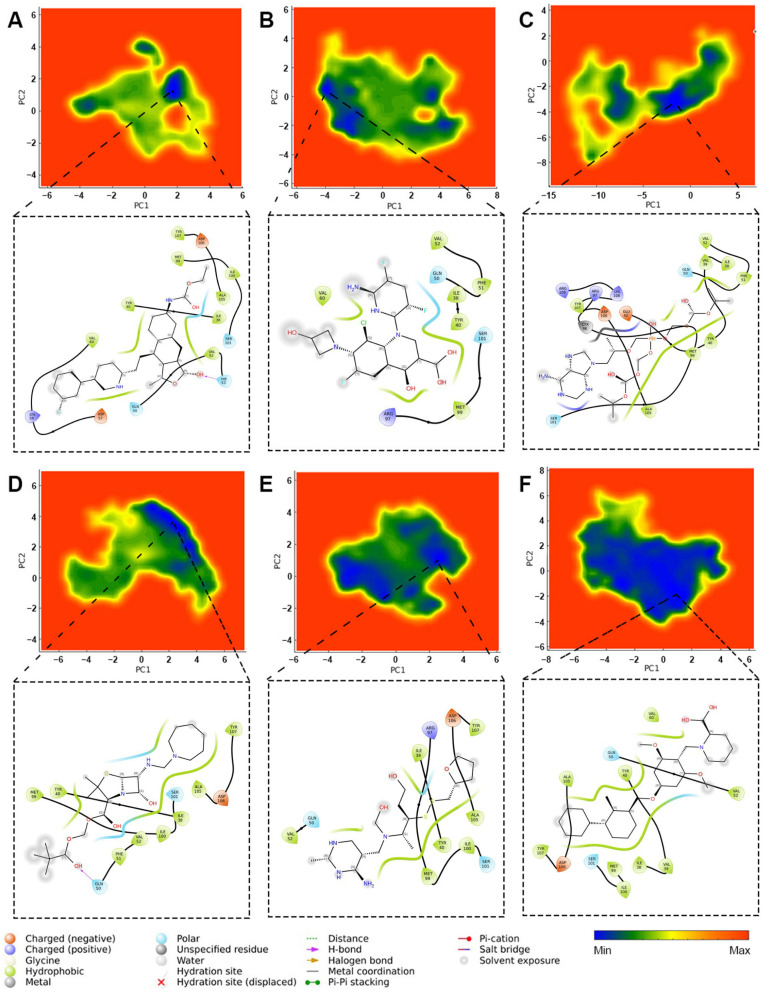
PCA plots and protein-ligand interaction diagrams at the lowest-energy conformations during long-term molecular dynamics simulations: (**A**) vorapaxar, (**B**) delafloxacin, (**C**) tenofovir disoproxil, (**D**) pivmecillinam, (**E**) fursultiamine, and (**F**) BMS-1.

Principal component analysis indicated that PC1 and PC2 mainly determined the distribution of binding free energy during the 150 ns molecular dynamics simulation. As shown in Figure 12, large blue-shaded areas signify lower-energy states, while red-shaded areas indicate higher-energy states. The PD-L1 inhibitor BMS-1 consistently occupied a lower-energy state across most principal component coordinates, implying that fluctuations in these components did not significantly influence its strong binding to PD-L1. Fursultiamine followed a similar trend, demonstrating a relatively narrow activity basin and maintaining a lower-energy state in most principal component coordinates.

Pivmecillinam and delafloxacin were found at intermediate energy levels, lacking broad anomalous activity basins, which suggests stable performance within the overall system. On the other hand, vorapaxar and pivmecillinam showed less favorability in this analysis, revealing frequent fluctuations along principal component coordinates and a notable absence of distinct clustering in lower-energy states.

### 2.4. Lead Selection and Reference Comparison

Based on the data from the post-molecular dynamics analysis, descriptive statistical evaluations were performed on the 5 final candidate compounds, as shown in Tables S2 and S3. Each parameter from the MD analysis received a specific weight based on its significance for PD-L1 inhibition and structural performance. The binding affinity (ΔG), regarded as the most critical factor, was assigned the highest weight (w = 0.30). Parameters indicating molecular stability and interaction consistency—namely, root-mean-square deviation (RMSD), root-mean-square fluctuation (RMSF), and contact frequency—were given intermediate weights (w = 0.16). Conversely, hydrogen bonding, along with structural compactness measures like radius of gyration (R_g_) and solvent-accessible surface area (SASA), was deemed to have lesser influence in this study and received minor weights (w = 0.07–0.08). Following the weighted scoring process, pivmecillinam was identified as the leading candidate for this research.

A comparative analysis of pivmecillinam and the reference compound BMS-1 revealed several significant similarities. As shown in Table S2, although BMS-1 demonstrated a noticeably more favorable binding free energy, pivmecillinam was the sole compound among the five candidates that maintained a stable binding conformation while exhibiting affinity values close to the reference compound. This similarity was further supported by their binding orientations within the PD-L1 receptor. Figure S2 illustrates that pivmecillinam and BMS-1 exhibit similar binding poses, suggesting a recurring interaction mode with key segments from PD-L1. Additionally, molecular interaction profiling, depicted in Figure S3, confirmed that both compounds engage PD-L1 mainly through π-alkyl and π-π stacking interactions. Besides, the key residues from the protein involved in these interactions—ILE38, TYR40, and MET99—remained consistent for both compounds, emphasizing a shared mechanism of interaction with PD-L1.

## 3. Materials and Methods

### 3.1. Protein and Drug Structure Preparation

The three-dimensional structure of PD-L1 (PDB ID: 5O45) was obtained from the RCSB Protein Data Bank [39]. This structure was crystallized by Magiera-Mularz et al. in 2017 using X-ray diffraction at a resolution of 0.99 Å [40]. A total of 1031 FDA-approved drug structures were gathered from the DrugBank [41]. The protein’s PDB file was pre-cleaned to remove water molecules and unnecessary heavy atoms. All drugs were assigned appropriate hydrogen atoms and converted into PDBQT format using Open Babel for the initial docking process [42]. Before the molecular dynamics (MD) simulations, all drugs were assigned partial charges and transformed into MOL2 format using the Antechamber module in AMBERTools 24 under the AM1 bond charge correction method [43].

### 3.2. Large-Scale Pre-Screening Methodology

In this study, a large-scale MD simulation was conducted for all 1031 FDA drugs from the library. AutoDock Vina 1.1.2 provided the initial binding pose for PD-L1 and drug complexes [44]. The center of the grid box was set at 155.996 Å, 9.013 Å, and −15.662 Å for the X, Y, and Z axes, respectively, with a search area of a 30 Å cubic box. The validation of grid box coordinates and size was performed using web-based services PrankWeb and CASTp [45,46].

Pre-screening of the 1031 FDA drugs was applied equilibration protocols, followed by the production phase [47]. A total of 2000 cycles of minimization, consisting of 1000 cycles of the steepest descent method plus another 1000 cycles of the gradient phase, allowed the complex system to reach a relatively relaxed state with all collisions released. A 30 ps heating phase under NVT conditions raised the system temperature from 100 K to 310 K, and a 500 ps mini-NPT phase smoothly transitioned the system into the next step. All FDA-approved drugs ultimately underwent a 10 ns production phase to assess their short-term dynamic stabilities and generate their initial binding free energies to score the entire drug library.

### 3.3. Reagents and Cell Culture

RPMI-1640 medium, along with a penicillin/streptomycin solution and fetal bovine serum (FBS), were acquired from Gibco BRL (Grand Island, NY, USA), while 3-(4,5-dimethylthiazol-2-yl)-2,5-diphenyltetrazolium bromide (MTT) was sourced from Sigma-Aldrich Corp. (St. Louis, MO, USA). The A549 cells (KCLB, Seoul, Republic of Korea) were cultured in RPMI enriched with streptomycin (100 μg/mL), penicillin (100 U/mL), and heat-inactivated FBS (10%) at 37 °C in a 5% CO_2_ cabinet [48].

### 3.4. MTT Assay Methodology

Cell viability was assessed through an MTT assay. In this process, 100 µL of A549 cells (1 × 10^4^) was exposed to the drug for 24 h [49]. Afterward, the cell suspension was combined with MTT solution (5 mg/mL) and incubated at 37 °C for an additional 4 h. Once the supernatant was discarded, the insoluble formazan was dissolved in dimethyl sulfoxide. Finally, the optical density of the 96-well culture plate was evaluated with a microplate reader at 540 nm [50].

### 3.5. Advanced Long-Term MD Simulations

Leading drugs, recognized for their top-tier pre-screening scores, engaged in focused and long-term molecular dynamics studies. Advanced molecular dynamic simulation model setup details followed protocols described by Jiang et al. in 2025 with minor adjustments [51]. The minimization period was extended to 5000 cycles, while both the NVT and NPT periods were increased to 1 ns to enhance stability. The production phase was further lengthened to 150 ns, consisting of 100 ns of stabilization dynamics and 50 ns of the analysis phase, resulting in 15,000 frames of trajectory.

### 3.6. Binding Free Energy Calculations

In this study, the MM-PBSA module of CPPTRAJ in AMBERTool 24 served as a crucial scoring method for ranking the FDA library [52]. Both the GB and PB models were adjusted to evaluate the binding affinities of these drugs to PD-L1. The calculation framework advised by the AMBER suite was applied to evaluating binding free energies [53]. This approach combines vacuum-phase binding energies with solvation energy differences to derive the overall binding free energy.

According to Equation (1), the vacuum-phase binding energy is determined by combining the molecular mechanics energy and the entropic contribution:

(1) $${∆G}_{vacuum}=E_{MM}-T\times \Delta S$$

where *E_MM_* represents the molecular mechanics energy, *T* denotes the temperature in Kelvin, and *S* signifies the entropic contribution. Equation (2) addresses the solvation-phase binding energy, computed by integrating the difference in dielectric constants between vacuum and solvent environments with a hydrophobic contribution:

(2) $${∆G}_{solv}=G_{electrostatic,\epsilon =80}-G_{electrostatic,\varepsilon =1}+{\Delta G}_{hydrophobic}$$

where *G_electrostatic_*_,_*_ϵ_*_=80_ indicates the solvent’s dielectric constant, *G_electrostatic_*_,_*_ϵ_*_=1_ represents the vacuum’s dielectric constant, and Δ*G_hydrophobic_* refers to hydrophobicity contribution. The final binding free energy is then obtained by summing the total vacuum-phase energy and the solvation adjustments, ensuring a comprehensive evaluation of ligand-receptor interactions as shown in Equation (3):

(3) $${\Delta G}_{TOTAL}={\Delta G}_{vacuum}+{\Delta G}_{solv}$$

### 3.7. Principal Component Analysis Methodology

Principal component analysis (PCA) was conducted using the post-MD analysis module in AMBERTools24 [52]. The covariance matrix was created by analyzing the fluctuations of all alpha carbon atoms in residues 1 to 130 throughout the simulation. By diagonalizing this matrix, a set of eigenvalues and eigenvectors were obtained, from which the first two principal components, referred to as PC1 and PC2, were extracted. Each frame of the binding free energy data from the MM-PBSA study, totaling 15,000 frames, was mapped onto PC1 and PC2 to identify the primary collective motions in relation to the corresponding free energy profiles.

## 4. Conclusions

The findings highlight the potential of repurposing existing drugs to inhibit PD-L1, contributing to advancements in immuno-oncology. Among the analyzed candidates, pivmecillinam emerged as the most promising, supported by its notable cytotoxic effects in cell-based assays and favorable outcomes in advanced molecular dynamics simulations. Its binding performance was comparable to that of the established PD-L1 inhibitor BMS-1, indicating a strong and stable interaction with the target protein. This study highlights the efficiency of combining computational and experimental approaches for accelerating drug discovery and exploring novel therapeutic options.

## Figures and Tables

**Table 1:** Major donor and acceptor residues from PD-L1 form hydrogen bonds with FDA-approved compounds and BMS-1.

H-Donor	H-Acceptor	Fraction	Total Fraction
Vorapaxar	TYR40	8.68%	15.35%
ARG109	Vorapaxar	6.67%	
Delafloxacin	MET99	7.54%	14.02%
ARG97	Delafloxacin	6.48%	
Tenofovir Disoproxil	TRY107	3.52%	28.67%
ARG109	Tenofovir Disoproxil	25.15%	
Pivmecillinam	MET99	2.73%	8.04%
GLN50	Pivmecillinam	5.31%	
Fursultiamine	MET99	5.08%	10.03%
SER101	Fursultiamine	4.95%	
BMS-1	GLN50	1.62%	5.28%
GLN50	BMS-1	3.66%	

**Table 2:** MM-PBSA binding free energy (kcal/mol) and energy component breakdown for leading FDA-approved compounds and BMS-1.

	Vorapaxar	Delafloxacin	Tenofovir Disoproxil	Pivmecillinam	Fursultiamine	BMS-1
VDW	−23.92 ± 4.00	−21.07 ± 3.62	−28.31 ± 5.40	−26.07 ± 4.46	−25.58 ± 3.78	−33.01 ± 4.12
EEL	−7.65 ± 6.85	17.89 ± 14.61	−9.19 ± 31.93	−2.85 ± 3.75	−3.76 ± 4.06	−5.15 ± 4.54
EGB	16.90 ± 6.71	−6.52 ± 14.77	24.10 ± 29.73	14.22 ± 4.12	13.42 ± 4.30	14.38 ± 4.06
ESURF	−3.31 ± 0.46	−2.67 ± 0.45	−4.15 ± 0.73	−3.32 ± 0.54	−3.30 ± 0.45	−3.92 ± 0.48
ΔG_gas_	−31.56 ± 9.17	−3.18 ± 15.61	−37.50 ± 34.54	−28.92 ± 5.76	−29.35 ± 5.33	−38.16 ± 6.38
ΔG_solv_	13.59 ± 6.47	−9.19 ± 14.77	19.95 ± 29.26	10.90 ± 3.95	10.12 ± 4.18	10.46 ± 3.97
ΔG_TOTAL_	−17.97 ± 3.88	−12.37 ± 4.00	−17.55 ± 7.50	−18.01 ± 3.94	−19.23 ± 3.94	−27.70 ± 4.44

## Data Availability

Data are contained within the article.

## Supplementary Materials

The following supporting information can be downloaded at: https://www.mdpi.com/article/10.3390/ijms26104497/s1.
