# Supplementary material for: Molecular Dynamics-Guided Repositioning of FDA-Approved Drugs for PD-L1 Inhibition with In Vitro Anticancer Potential
Source: Int J Mol Sci. 2025 May 8;26(10):4497. doi: 10.3390/ijms26104497 (PMC12110937; doi:10.3390/ijms26104497)
Supplement: Supplementary file 1 [file ijms-26-04497-s001.zip › ijms-3595778-supplementary.pdf]

Supplementary Materials

Table S1. List of drugs discovered during pre-screening, including their CAS numbers, indications, molecular weights, and binding free energies.

Compound ID	Compound Name	CAS No.	Original Indication	M.W. (g/mol)	Binding Energy (kcal/mol)	
					GB-Model	PB-Model
24	Ambrisentan	177036-94-1	Pulmonary HTN	378.41	−40.7403	−37.7370
32	Amisulpride	53583-79-2	Schizophrenia	369.48	−40.5234	−35.1729
85	Aztreonam	78110-38-0	Antibiotic	435.42	−51.3232	−45.4938
115	Brigatinib	1197953-54-0	NSCLC (Oncology)	584.10	−43.6536	−43.8248
162	Cefmetazole	56796-20-4	Antibiotic	471.50	−46.6107	−43.3766
164	Cefotaxime	63527-52-6	Antibiotic	455.46	−43.4932	−39.5092
176	Ceftriaxone	73384-59-5	Antibiotic	554.58	−44.0133	−38.3597
179	Cephaloglycin	3577-01-3	Antibiotic	405.40	−45.3173	−39.9266
201	Cilastatin	82009-34-5	DHP-1 inhibitor	358.50	−55.0174	−50.7472
209	Clarithromycin	81103-11-9	Antibiotic	747.96	−29.5836	−42.0297
230	Cocarboxylase	154-87-0	Vitamin B1 deficiency	460.77	−43.3220	−39.7838
256	Decamethonium	156-74-1	Neuromuscular block	258.49	−46.2723	−43.2896
259	Delafloxacin	189279-58-1	Antibiotic	440.80	−52.5564	−43.506
312	Dolutegravir	1051375-16-6	HIV	419.38	−44.6386	−36.3776
353	Ertugliflozin	1210344-57-2	Type 2 diabetes	436.90	−40.5190	−35.6058
408	Flutemetamol	637003-10-2	Imaging	274.32	−40.1964	−34.0800
410	Formoterol	73573-87-2	Asthma/COPD	344.40	−41.8816	−39.7944
419	Fursultiamine	804-30-8	Vitamin B1 deficiency	398.50	−41.2444	−41.7590
437	Gemifloxacin	175463-14-6	Antibiotic	389.40	−44.2295	−38.3693
457	Hexafluronium	4844-10-4	Neuromuscular block	502.70	−29.2500	−41.3505
491	Inositol Nicotinate	6556-11-2	Lipid lowering	810.70	−40.9897	−54.3613
500	Iopanoic Acid	96-83-3	Radiocontrast	570.93	−41.2931	−34.3117
625	Metocurine	5152-30-7	Neuromuscular block	652.80	−38.4597	−46.1312
662	Nedocromil	69049-73-6	Asthma	371.30	−42.1558	−37.1411
693	Olsalazine	6054-98-4	Ulcerative colitis	346.20	−40.6433	−27.8788
718	Palbociclib	571190-30-2	Breast cancer (Oncology)	447.53	−51.1475	−41.6877
739	Perazine	84-97-9	Antipsychotic	339.50	−42.1626	−40.1877
751	Phenoxymethylpenicillin	87-08-1	Antibiotic	350.38	−42.5230	−38.5515
780	Pivmecillinam	32886-97-8	Antibiotic	439.60	−46.0489	−48.8358
783	Plazomicin	1154757-24-0	Antibiotic	592.69	−42.4499	−43.6668
846	Roxithromycin	80214-83-1	Antibiotic	837.06	−33.7584	−45.9881
871	Succinylcholine	306-40-1	Neuromuscular block	290.40	−47.1479	−38.7020
907	Telaprevir	402957-28-2	Hepatitis C	679.80	−31.5787	−43.6992
912	Tenofovir Disoproxil	201341-05-1	HIV, Hepatitis B	519.40	−50.4016	−51.4527
913	Terbinafine	91161-71-6	Antifungal	291.43	−46.8543	−39.1572
941	Tolbutamide	64-77-7	Type 2 diabetes	270.35	−42.4302	−33.6972
948	Torasemide	56211-40-6	Diuretic	348.41	−46.1315	−42.6929
961	Trifluoperazine	117-89-5	Antipsychotic	407.50	−37.1022	−40.7349
985	Uridine Triacetate	4105-38-8	Hereditary orotic aciduria	370.31	−40.1656	−37.9846
1005	Vitamin A	68-26-8	Vitamin deficiency	286.45	−44.2035	−33.0082
1006	Vorapaxar	618385-01-6	Antiplatelet	492.60	−43.8983	−39.9600

Table S2. Molecular dynamics stability and interaction metrics for each repositioned compound compared to the reference PD-L1 inhibitor BMS-1.

Compound	RMSD (Å)		RMSF (Å)		R_g (Å)		SASA (Å²)		H-bonds		Contacts		ΔG (kcal/mol)	
	Mean ± SD	Rank	Mean ± SD	Rank	Mean ± SD	Rank	Mean ± SD	Rank	Mean ± SD	Rank	Mean ± SD	Rank	Mean ± SD	Rank
Vorapaxar	2.103 ± 0.102	1	1.272 ± 0.570	1	16.223 ± 0.307	5	7573.270 ± 178.519	5	0.389 ± 0.639	3	15.980 ± 18.246	5	-17.972 ± 3.881	3
Delafloxacin	2.261 ± 0.193	4	1.803 ± 0.708	5	16.073 ± 0.285	3	7397.139 ± 187.047	3	0.580 ± 0.836	2	17.505 ± 27.792	4	-12.372 ± 4.003	5
Tenofovir Disoproxil	2.181 ± 0.136	2	1.389 ± 0.495	3	15.698 ± 0.204	2	7405.879 ± 183.538	4	0.870 ± 1.118	1	26.445 ± 32.574	3	-17.547 ± 7.502	4
Pivmecillinam	2.238 ± 0.128	3	1.320 ± 0.591	2	15.626 ± 0.135	1	7141.516 ± 226.855	1	0.233 ± 0.532	5	31.278 ± 26.891	2	-18.013 ± 3.944	2
Fursultiamine	2.313 ± 0.180	5	1.586 ± 0.616	4	16.168 ± 0.250	4	7392.330 ± 208.627	2	0.359 ± 0.649	4	49.901 ± 37.331	1	-19.232 ± 3.943	1
BMS-1	2.149 ± 0.123	ref	1.591 ± 0.604	ref	16.527 ± 0.668	ref	7571.889 ± 206.471	ref	0.211 ± 0.508	ref	29.283 ± 21.611	ref	-27.702 ± 4.441	ref

Table S3. Weighted composite scoring of the post-MD analysis and overall ranking of the five PD-L1 inhibitor candidates.

Compound	RMSD		RMSF		R_g		SASA		H-bonds		Contacts		ΔG		Overall Score	Overall Rank
	Rank	Weighted (w=0.16)	Rank	Weighted (w=0.16)	Rank	Weighted (w=0.07)	Rank	Weighted (w=0.07)	Rank	Weighted (w=0.08)	Rank	Weighted (w=0.16)	Rank	Weighted (w=0.30)		
Vorapaxar	1	0.16	1	0.16	5	0.35	5	0.35	3	0.24	5	0.80	3	0.90	2.96	3
Delafloxacin	4	0.64	5	0.8	3	0.21	3	0.21	2	0.16	4	0.64	5	1.50	4.16	5
Tenofovir Disoproxil	2	0.32	3	0.48	2	0.14	4	0.28	1	0.08	3	0.48	4	1.20	2.98	4
Pivmecillinam	3	0.48	2	0.32	1	0.07	1	0.07	5	0.40	2	0.32	2	0.60	2.26	1
Fursultiamine	5	0.80	4	0.64	4	0.28	2	0.14	4	0.32	1	0.16	1	0.30	2.64	2

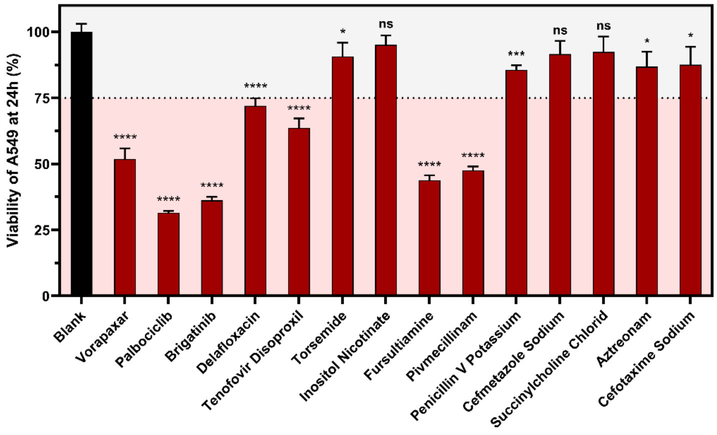

Figure S1. Results of the primary in vitro assay conducted on 14 commercially available compounds, highlighting candidates that exhibited cancer cell viability below 75% after 24 hours of treatment; **** $p < 0.0001$, *** $p < 0.001$, * $p < 0.05$, and ns indicates $p > 0.05$, based on Welch's t-test, with the dashed line representing 75% cell viability.

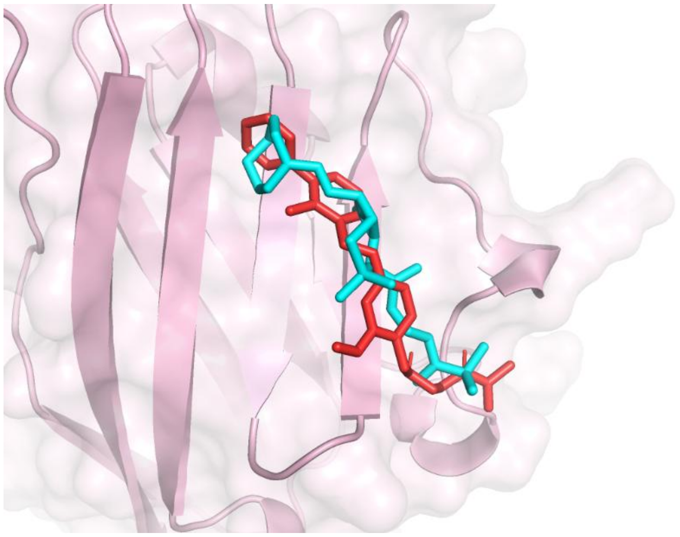

Figure S2. Binding conformations of pivmecillinam (cyan) and the reference inhibitor BMS-1 (red) within the PD-L1 binding pocket.

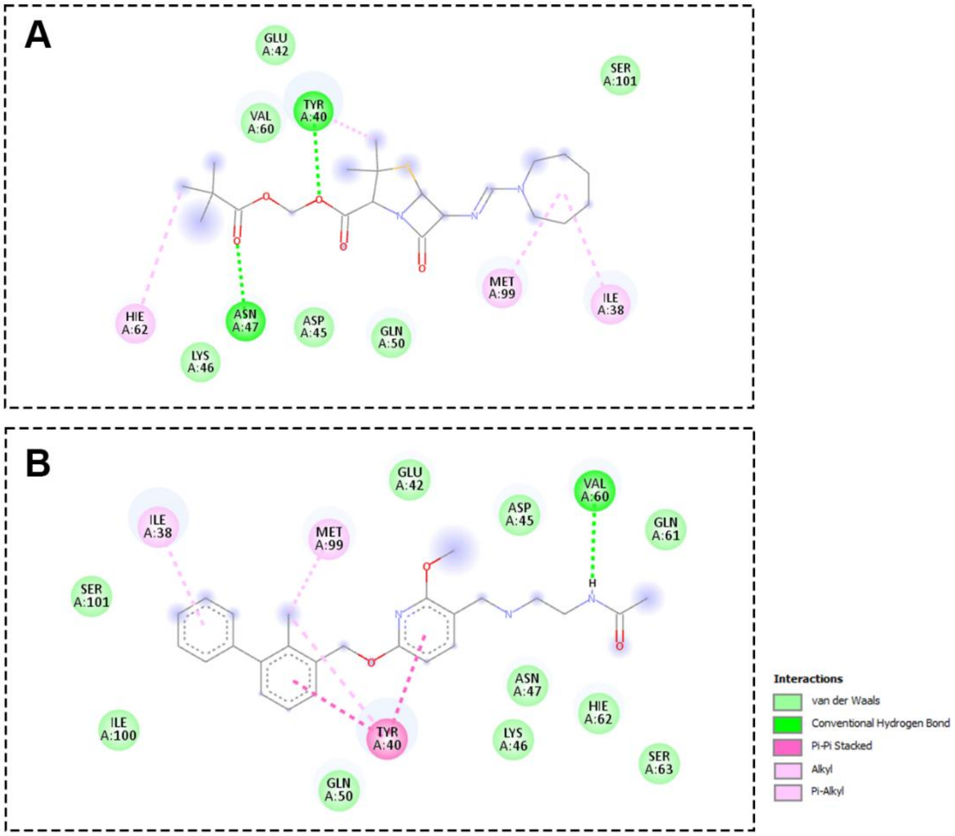

Figure S3. Molecular interaction analysis of PD-L1 with (A) pivmecillinam and (B) BMS-1.
